# Cocaine-Associated Myocardial Infarction: Should They All Be Stented?

**DOI:** 10.1155/2011/347806

**Published:** 2011-07-19

**Authors:** Sazzli Kasim, Ronan O'Donabhain, Eugene Mcfadden

**Affiliations:** Department of Cardiology, Cork University Hospital, Cork, Ireland

## Abstract

Cocaine use is a known cause of chest pain and acute myocardial infarction and frequently leads to cardiac catheterization procedure. The treatment of cocaine-related acute coronary syndromes presents unique challenges because a variety of mechanisms including atherosclerotic plaque rupture, platelet activation, and coronary vasospasm may contribute to the pathogenesis. Our case highlights important considerations taken in dealing with this acute scenario

## 1. Introduction

Cocaine represents the second commonest substance abuse in the USA with an increasing number of visits to the Emergency Department. Cocaine-associated myocardial infarction (CAMI) has an incidence varying between 0.7 and 6% of all MIs, with an average age of 38 years old [1].

## 2. Case Report

**Figure 1:**
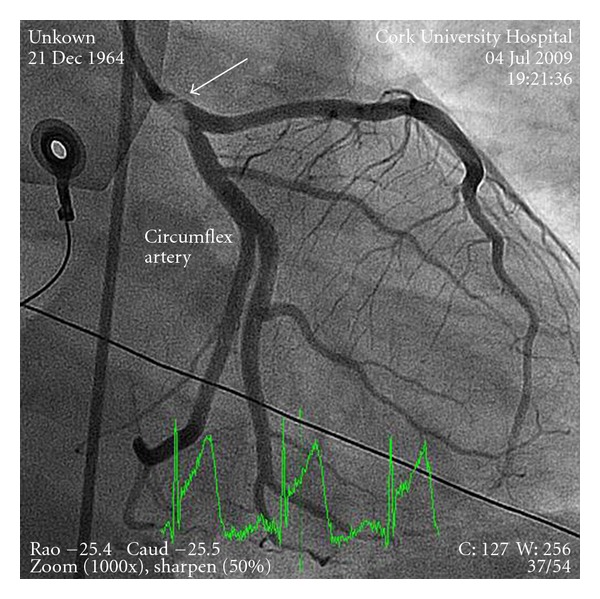
Right anterior oblique with caudal angulation showing the left main coronary artery full of thrombus (arrow), circumflex, and left anterior descending artery.

**Figure 2:**
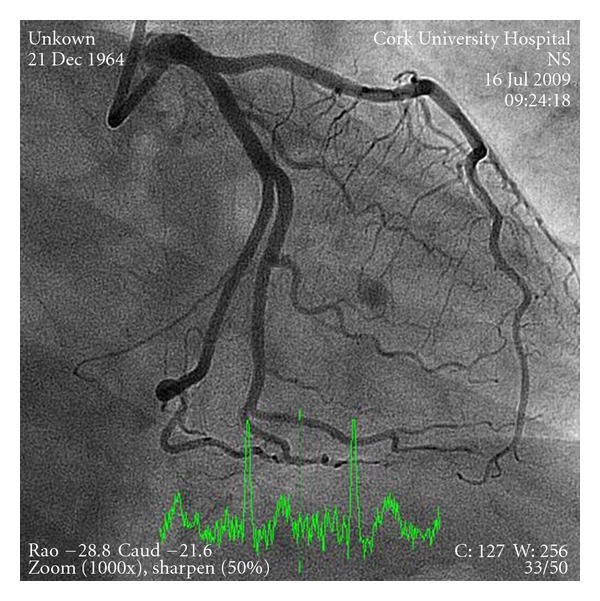
Similar projections showing resolution of thrombus with normal underlying coronary arteries.

A 46-year-old male presented to our center with sudden onset retrosternal chest pain lasting for 4 hours. He was haemodynamically stable with an electrocardiograph revealing a 3 mm ST segment elevation myocardial infarction (STEMI) in the inferior leads. He is a smoker and smoked cocaine the night before, 8 hours prior to symptoms. No other cardiac risk factors were identified. He was treated with standard ACS therapy including oral acetylsalicylic acid 300 mg and clopidogrel 600 mg loading dose. Emergent angiography revealed thrombus in the left main coronary artery partially occluding a dominant left circumflex with TIMI III flow (Figure 1). The coronaries were otherwise normal, with good left ventricular systolic function. The large thrombus burden in the left main coronary artery posed a strategic dilemma. We opted to treat with intravenous abciximab (a glycoprotein IIb/IIIa inhibitor), bolus, and infusion along with 7 days of therapeutic dose of enoxaparin, acetylsalicylic acid 75 mg daily, and clopidogrel 75 mg daily. The patient had an unremarkable clinical course. Repeat angiography twelve days later with IVUS guidance demonstrated complete resolution of thrombus (Figure 2).

## 3. Discussion

Cocaine potentiates thrombus formation. It causes coronary vasoconstriction by simultaneous stimulation of alpha-adrenergic receptors, increased endothelin-1 production, and reduction in nitric oxide levels [2]. Thrombus formation is further enhanced by stimulation of platelet activators and altered balance between procoagulant and anticoagulant factors [3].

The optimal management of CAMI is still unknown. In our case, several therapeutic options merit discussion. Primary PCI is preferred to lysis in STEMI associated with cocaine [1]. Patients intoxicated with cocaine may have a higher risk of aortic dissection and intracerebral haemorrhage from underlying aortopathy and significantly raised blood pressure, a situation where thrombolysis may prove fatal. Coronary stenting in CAMI involving the left main coronary is an unproven strategy. As recidivism is high in this cohort, compliance to dual antiplatelet therapy may vary, resulting in a higher risk of stent thrombosis [4] which carries a significant mortality risk. Thromboaspiration devices have been shown to improve outcomes but were avoided here to prevent further clot embolisation downstream. The use of GpIIb/IIIa inhibitors has been described with good effect [5]. In our case, a conservative approach with GpIIb/IIIa inhibitor along with dual antiplatelet usage allowed for intrinsic fibrinolysis as well as an accurate psychosocial and drug compliance assessments. Repeat angiography should be performed if hemodynamic deterioration occurs and to demonstrate vessel patency. Secondary prevention and avoidance of further cocaine use should be advocated in all CAMI patients. We propose withholding the “oculo-stent” reflex where possible to allow for complete assessment of the individual patient and the avoidance of unnecessary exposure to stent related co-morbidities in the future.
